# Digitale Gesundheitsanwendungen auf Rezept – oder auch nicht? Experteninterviews zu Verordnung und Selbstantrag von DiGA

**DOI:** 10.1055/a-2800-1191

**Published:** 2026-06-03

**Authors:** Godwin Giebel, Klemens Höfer, Pascal Raszke, Jürgen Wasem, Anna Bußmann, Sarah Schlierenkamp, Michael Minor, Carsten Volland, Udo Schneider, Enno Maaß, Josepha Katzmann, Carina Abels

**Affiliations:** 1 Lehrstuhl für Medizinmanagement, Universität Duisburg-Essen, Essen, Germany; 2 Essener Forschungsinstitut für Medizinmanagement GmbH, Essen, Germany; 3 Versorgungsmanagement, Techniker Krankenkasse, Hamburg, Germany; 4 Deutsche Psychotherapeuten Vereinigung (DPtV), Berlin, Germany

**Keywords:** Digitale Gesundheitsanwendung, DiGA, Psychotherapie, Apps, Digitale Therapie, Digital Health Application, mHealth App, prescription, psychotherapy, digital care

## Abstract

**Hintergrund:**

In Deutschland kommen seit 2020 Digitale Gesundheitsanwendungen (DiGA) im Rahmen der Behandlung psychischer Erkrankungen zum Einsatz. DiGA können von gesetzlich Versicherten über zwei verschiedene Zugangswege erlangt werden. Entweder können sie durch einen Arzt oder Psychotherapeuten verordnet oder bei gegebener Indikation direkt bei der Krankenkasse beantragt werden. Das Ziel der vorliegenden Studie ist es, die verschiedenen Zugangswege insbesondere hinsichtlich der tatsächlichen Umsetzung genauer zu betrachten.

**Methodik:**

Es wurden leitfadengestützte Interviews mit Experten durchgeführt, um die aktuelle Versorgungssituation mit DiGA im Bereich psychischer Erkrankungen zu untersuchen. Bei den Experten handelte es sich um Vertreter von Patienten, Leistungserbringenden, Krankenkassen und DiGA-Herstellern. Die Interviews wurden online durchgeführt, aufgezeichnet und transkribiert. Die Transkripte wurden mit Hilfe der Software MAXQDA einer qualitativen Inhaltsanalyse in Anlehnung an Kuckartz unterzogen.

**Ergebnisse:**

Es wurden 13 Experteninterviews geführt. Nach inhaltlicher Analyse ergaben sich die drei Hauptthemen: 1) Relevanz der direkten Genehmigung bei der Krankenkasse, 2) Umsetzung der direkten Genehmigung durch die Krankenkassen und 3) Vor- und Nachteile des direkten Zugangs. Während der direkte Zugang über die Krankenkassen quantitativ eher eine untergeordnete Rolle spielt, birgt er auch mögliche Vorteile wie einen niederschwelligen Zugang oder eine größere Patientenautonomie. Nachteile wurden insbesondere in einer Verlagerung der Therapiehoheit von Leistungserbringern auf die Krankenkassen, das Fehlen einer umfassenden Therapieüberwachung, unzureichende Diagnosestellungen ohne oder mit nicht ausreichender Berücksichtigung von Kontraindikationen sowie einem Mehraufwand für die Krankenkassen gesehen.

**Schlussfolgerung:**

Mit der Möglichkeit zur Beantragung von GKV-finanzierten Leistungen direkt bei den Krankenkassen wurde im deutschen Gesundheitssystem ein neuer Weg eingeschlagen. Während es Argumente für sowie wider den Selbstantrag gibt, ist die aktuelle Umsetzung zu hinterfragen. Durch die Notwendigkeit eines aktuellen Indikationsnachweises sowie gegebenenfalls durch den nötigen Ausschluss von Kontraindikationen müssen Patienten in der Regel den Arzt bzw. Psychotherapeuten ohnehin aufsuchen, was die möglichen Vorteile deutlich einschränkt. Unter den aktuellen Rahmenbedingungen ist der Weg der Verordnung meist mit weniger Problemen verbunden.

## Abstract (en)

**Introduction:**

In Germany, Digital Health Applications (DiGA) can be prescribed for mental illnesses since 2020. Patients with statutory health insurance can access DiGA in two different ways. They can either be prescribed by a physician or psychotherapist or patients can request them directly from their health insurance company. The aim of the present study was to shed light on the different ways of access especially with regard to their actual implementation.

**Methods:**

Semistructured interviews were conducted with experts to investigate the current care situation with DiGA in the context of mental illnesses. The experts were representatives of patients, physicians and psychotherapists, statutory health insurance companies and DiGA manufacturers. The interviews were conducted online, recorded and transcribed. A qualitative content analysis according to Kuckartz was performed with MAXQDA.

**Results:**

A total of 13 expert interviews were conducted. Content analysis revealed three main topics: 1) relevance of direct authorization by the health insurance company, 2) implementation of direct authorization by the health insurance company, and 3) advantages and disadvantages of direct access. While direct access via the health insurance company played a minor role quantitatively, it has potential advantages such as a low threshold access or a greater patient autonomy. Disadvantages were seen in the transfer of therapeutic sovereignty from physicians and psychotherapists towards the health insurance companies. Problems were also seen in the lack of comprehensive therapy monitoring, inadequate diagnosis without or with inadequate consideration of contraindications, and additional effort on the part of health insurance companies.

**Conclusion:**

The direct application for statutory health insurance-funded services from health insurance companies marks a new approach in the German health care system. While there are good arguments for and against patient self-referral, the current implementation needs to be scrutinized. Because of the need for a current indication and the possible exclusion of contraindications, patients usually have to visit a doctor or psychotherapist anyway, which significantly limits the benefits. Under the current conditions, the way of prescription is usually associated with fewer problems.

## Einleitung

Mit der Listung der ersten Digitalen Gesundheitsanwendung (DiGA) im September 2020 im DiGA-Verzeichnis durch das Bundesinstitut für Arzneimittel und Medizinprodukte (BfArM) erhielten Versicherte der gesetzlichen Krankenversicherung (GKV) erstmals die Möglichkeit, bei gestellter Diagnose oder Verordnung auf dieses neue Versorgungsangebot zurückzugreifen. Seitdem stieg die Anzahl an verfügbaren DiGA bis heute (Dezember 2025) auf 57 an 1 . Auffällig ist der mit 29 DiGA sehr hohe Anteil an Apps, die auf Erkrankungen der Psyche abzielen. Das Krankheitsspektrum, im Bereich „Psyche“, beinhaltet unter anderem Depression, Angst- und Panikstörungen, soziale Phobien, Nichtorganische Insomnie und Suchterkrankungen 1 . In der Regel dienen DiGA zur Behandlung psychischer Erkrankungen dazu, Elemente und Methoden der kognitiven Verhaltenstherapie im Rahmen eines störungsspezifischen Online-Kurses für Patienten zur selbstständigen oder therapiebegleitenden Bearbeitung anzubieten 2 .

Neben der absoluten Anzahl an verfügbaren DiGA im Bereich der psychischen Erkrankungen ist auch der Marktanteil mit 30% aller ausgegebenen DiGA der größte. Die Nutzer in dem Indikationsgebiet sind überwiegend weiblich (69%). Ihr mittleres Alter liegt bei 42 Jahren 3 .

Um eine DiGA nutzen zu können, gibt es für Patienten grundsätzlich die Möglichkeit zwischen 2 verschiedenen Zugangswegen zu wählen (§ 33a SGB V): entweder die Verordnung durch den behandelnden Arzt beziehungsweise Psychotherapeuten oder bei Nachweis der medizinischen Indikation, für die die DiGA bestimmt ist, direkt über die Krankenkasse 4 .

Im Bereich psychischer Erkrankungen erfolgt der Zugang zur DiGA meist durch eine Verordnung von Ärzten oder Psychotherapeuten. So werden im Kontext von Depressionen 48% der DiGA über Hausärzte, 34% über weitere Ärzte und 10,9% über psychologische Psychotherapeuten verordnet. Im Bereich der Angststörung verordnen Hausärzte 38%, sonstige Ärzte 30,6% und psychologische Psychotherapeuten 22,7% aller DiGA in diesem Indikationsgebiet. Jeweils der Rest (Depression: 6,2%, Angststörung: 7,8%) wird direkt bei den Krankenkassen beantragt 5 .

Da der direkte Zugangsweg über die Krankenkassen bisher eine eher untergeordnete Rolle spielte 3 und von verschiedenen Stakeholdern kontrovers diskutiert wird 6 7 , sollen im Rahmen der vorliegenden Untersuchung die folgenden Fragestellungen beantwortet werden: 1) Welche Relevanz hat die direkte Genehmigung der Krankenkassen, 2) Wie wird diese durch die Krankenkassen umgesetzt und 3) Welche Vor- und Nachteile bringt dieser Zugangsweg mit sich?

Die vorliegende Untersuchung ist Teil des Forschungsprojekts „Die Umsetzung von Potenzialen Digitaler Gesundheitsanwendungen (DiGA) in der ambulanten Versorgung psychischer Erkrankungen“ (DiGAPsy). Dieses untersucht die Einbindung digitaler Angebote in die ambulante Versorgung von Menschen mit psychischen Erkrankungen 8 . Das Projekt wird durch den Innovationsausschuss des Gemeinsamen Bundesausschusses gefördert (Förderkennzeichen: 01VSF22029). Der vorliegende Artikel zielt speziell auf den Zugangsweg zu DiGA über Genehmigung durch Krankenkassen ab. Die Ergebnisse dienten unter anderem der Konzeptionierung einer quantitativen Befragung von Patienten und Leistungserbringern.

## Methodik

Es wurden leitfadengestützte Online-Experteninterviews durchgeführt, um die aktuelle Versorgungssituation mit DiGA im Bereich psychischer Erkrankungen zu untersuchen. Der Artikel und insbesondere die Methodik wurde mit Hilfe der 32-Item Consolidated Criteria for Reporting Qualitative Research (COREQ) Checklist 9 angefertigt und geprüft.

### Ethische Aspekte

Für das übergeordnete Forschungsprojekt wurde durch die Ethikkommission der medizinischen Fakultät der Universität Duisburg-Essen ein Ethikvotum beraten und genehmigt (23-11209-BO). Im Rahmen der Interviews wurden weder sensible (z. B. krankheitsbezogene) noch persönliche (z. B. Alter, Geschlecht) Daten erhoben. Vor der Durchführung der Gespräche wurden die Teilnehmer über das Thema, das Ziel und das Vorgehen der Interviews informiert. Die E-Mail enthielt weiterhin Informationen zum Datenschutz und darüber, dass die Gespräche aufgezeichnet und transkribiert werden würden, um eine anschließende Datenanalyse durchführen zu können. Weiterhin wurden die Teilnehmer darüber informiert, dass sie die Gespräche jederzeit, ohne Angabe von Gründen abbrechen könnten, ohne, dass ihnen ein Nachteil entstünde. Alle interviewten Experten stimmten vor den Gesprächen einer Teilnahme in Form eines digital unterschriebenen Dokuments explizit zu. Im Anschluss an die Transkription wurden die Videoaufzeichnungen gelöscht. Durch das Löschen der Aufzeichnungen und eine Pseudonymisierung aller identifizierenden Angaben (z. B. Name, Institution, etc.) im Rahmen der Transkription sind Rückschlüsse auf befragte Personen nicht mehr möglich.

### Auswahl der Befragten

Es wurde nach Experten gesucht, die die Interessen von Leistungserbringern, Patienten, DiGA-Herstellern im Bereich psychischer Erkrankungen und Krankenassen abbilden. Nach einer Abstimmung mit dem Projekt-Konsortium wurden insgesamt 15 Institutionen kontaktiert; 2 davon lehnten eine Teilnahme an der Untersuchung ab. Bei der Auswahl der Krankenkassen wurde darauf geachtet, dass verschiedene Krankenkassenarten vertreten waren. Im Falle der Hersteller wurden sowohl solche mit mehreren DiGA, als auch kleinere Hersteller mit nur einer DiGA angesprochen. Weiterhin wurde darauf geachtet, dass sowohl Hersteller mit vorläufig, als auch mit dauerhaft zugelassenen DiGA vertreten waren. Bei den Leistungserbringern wurden Ärzte- sowie Psychotherapeutenkammern und Kassenärztliche Vereinigungen angesprochen, um einen breiten Überblick zu erlangen. Soziodemographische Eigenschaften der Teilnehmer wurden nicht berücksichtigt beziehungsweise erhoben, da die Untersuchung auf das davon unabhängige Expertenwissen abzielte.

### Rahmenbedingungen und Datenerhebung

Die Interviews wurden online über Microsoft Teams durchgeführt. Neben mindestens zwei der Moderatoren (KH, CA, FP) und den jeweiligen Interviewpartnern waren keine weiteren Personen anwesend. Alle drei Moderatoren haben einen wirtschaftswissenschaftlichen Hintergrund mit dem Schwerpunkt Medizinmanagement. Zum Zeitpunkt der Studiendurchführung waren sie als wissenschaftliche Mitarbeiter (KH, FP) bzw. Arbeitsgruppenleitung (CA) im Bereich Medizinmanagement angestellt. Zwei der Moderatoren sind männlich (KH, FP), eine Moderatorin ist weiblich (CA). Alle verfügten über vorherige Interviewerfahrung aus anderen Projekten. Die Interviews wurden audio-visuell aufgezeichnet und anschließend von studentischen Hilfskräften transkribiert. Während der Transkription wurde eine Pseudonymisierung vorgenommen. Keiner der Moderatoren hatte eine klare Haltung für oder gegen den Einsatz von DiGA. Es bestehen keine Interessenskonflikte. Zwei der Interviewer (CA und FP) kannten zwei der Interviewpartner bereits aus einem Interview bzw. Workshop im Rahmen eines vorherigen Innovationsfondsprojektes (01VFS20007).

Nach einer kurzen Vorstellung der Beteiligten wurden die Interviews mithilfe eines teilstrukturierten Leitfadens (nach behandelten Themen strukturiert vgl. Appendix 1) durchgeführt. Dieser beruhte auf Ergebnissen eines vorher durchgeführten Scoping Reviews 10 . Der Leitfaden wurde entsprechend der fachlichen Hintergründe der Teilnehmer leicht modifiziert. Teilnehmende wurden vor den Interviews über das Thema der Gespräche, nicht aber über die Inhalte des Leitfadens, informiert.

### Datenanalyse

Die Datenanalyse wurde in Anlehnung an Kuckartz 11 mit Hilfe von MAXQDA durchgeführt. Dabei handelte es sich konkret um eine strukturierende qualitative Inhaltsanalyse, bei der einzelne Teilschritte zusammengefasst wurden. Die primäre Datenanalyse fand im November 2023 statt. Im Zuge der Veröffentlichung wurden die Daten im November und Dezember 2024 final aufbereitet und strukturiert.

Vor Beginn der Datenauswertung wurden deduktive Kodes auf Basis der Themen des Fragebogens entwickelt (KH, CA). Während der initialen Kodierung (PR) wurden weitere induktive Kodes abgeleitet, um identifizierte (Sub-)Themen festzuhalten. In einer zweiten Runde wurden die Kodes qualitätsgesichert (CA). Dabei wurde insbesondere die Zuordnung einzelner Textstellen zu den Hauptkategorien und die abschließende Kodierung der deduktiven und induktiven Kodes hinsichtlich inhaltlicher Konsistenz überprüft. Im Zuge der Manuskripterstellung wurde die Systematik final adaptiert (GDG). Hinsichtlich der Kodierung der Zugangswege wurden die relevanten Aussagen in die 2 Bereiche Selbstantrag (Genehmigungsprozess) und Verschreibungsprozess aufgeteilt. Zu den finalen Ergebnissen wurde kein Feedback der Teilnehmer eingeholt.

## Ergebnisse

Insgesamt wurden 13 Interviews mit 14 Teilnehmenden geführt (vgl. Tab. 1 ). An den Interviews nahmen Vertreter der Leistungserbringenden (n=5), der Krankenkassen (n=4), der DiGA-Hersteller (aus dem Bereich psychischer Erkrankungen) (n=4) und der Patienten (n=1) teil.

**Table:** **Tab. 1** Übersicht über die geführten Interviews.

Nr.	Gruppe	Setting	Datum
1	Patienten	Einzelinterview	14.09.2023
2	Krankenkasse	Einzelinterview	10.10.2023
3	Krankenkasse	Einzelinterview	12.10.2023
4	DiGA-Hersteller	Einzelinterview	18.10.2023
5	Leistungserbringer	Einzelinterview	24.10.2023
6	DiGA-Hersteller	Einzelinterview	25.10.2023
7	Leistungserbringer	Einzelinterview	26.10.2023
8	Krankenkasse	Doppelinterview	30.10.2023
9	DiGA-Hersteller	Einzelinterview	31.10.2023
10	DiGA-Hersteller	Einzelinterview	02.11.2023
11	Leistungserbringer	Einzelinterview	02.11.2023
12	Leistungserbringer	Einzelinterview	07.11.2023
13	Leistungserbringer	Einzelinterview	07.11.2023

Es wurden keine soziodemografischen Charakteristika der Teilnehmer erhoben. Die Dauer der Interviews betrug zwischen 52 und 82 Minuten. Im Folgenden werden die Ergebnisse entsprechend der 3 zugrunde gelegten Fragestellungen präsentiert.

### Welche Relevanz hat die direkte Genehmigung der Krankenkasse?

Grundsätzlich ist der Selbstantrag entsprechend der Expertenaussagen der deutlich seltener genutzte Weg, um Zugang zu DiGA zu erhalten. Entsprechend der Aussagen der Vertreter von Krankenkassen und Psychotherapeuten wird dieser Weg in ca. 5 bis 11% der Fälle genutzt. Ohne konkrete Zahlen zu nennen, wird diese Aussage auch durch DiGA-Hersteller untermauert.

*„Unserer Erfahrung nach wird das sehr selten von Patienten gewählt als Weg, also es ist-, es spielt einfach keine besonders große Rolle in der Realität, das ist wichtig.“ (DiGA-Hersteller)*

### Wie wird die direkte Genehmigung durch die Krankenkassen umgesetzt?

#### Unterstützung der Krankenkassen bei der DiGA-Auswahl

Eine Beratung der Patienten im Kontext des Selbstantrags durch die Krankenkassen geschehe laut Herstellern und Krankenkassen zurückhaltend. Die Krankenkassen verwiesen auf Schwierigkeiten bei der Beratung unter anderem aufgrund fehlender medizinischer Kenntnisse zu Diagnosen und Kontraindikationen. Weiterhin wurde konstatiert, dass ihnen als Krankenkasse nur selten Testzugänge der Hersteller zur Verfügung gestellt würden und es darüber hinaus auch grundsätzlich schwierig sei, Unterschiede zwischen DiGA festzustellen und Versicherte sinnvoll zu beraten. Teilweise würde sich die Beratung auf einen Verweis auf das DiGA-Verzeichnis des BfArMs beschränken. Eine Kasse erklärte, dass ihnen der Einbezug der Leistungserbringenden wichtig sei:

*„[…] wir möchten, dass die DiGA in die Versorgung integriert wird. Das heißt, dass soll nicht für sich alleine laufen, deswegen sagen wir, unbedingt mit deinem Mediziner sprechen, mit dem behandelnden Arzt oder Psychotherapeuten, weil der das Ganze begleiten soll. Und der muss dann natürlich auch sagen, was hält der für dich da an der Stelle für richtig und muss dich da auch begleiten.“ (Krankenkasse)*

#### Ausgestaltung des Genehmigungsprozesses

Um eine DiGA über den Weg des Selbstantrags zu erhalten, ist der Antrag nicht formgebunden. Der Abgleich der Voraussetzungen aus dem DiGA-Verzeichnis (z. B. Diagnose) mit den vorliegenden Patienteninformationen geschieht in der Regel durch Sachbearbeiter, teilweise systemgestützt. Während das Alter und Geschlecht des Patienten einfach durch die Krankenkassen geprüft werden können, ist die Prüfung der Indikation und vorliegenden Kontraindikationen komplexer. Ein Indikationsnachweis kann über mehrere Wege erfolgen. In den Interviews wurden zum Beispiel Krankenhausaufenthalte, eine Arbeitsunfähigkeitsbescheinigung oder die Teilnahme an Disease-Management-Programmen als mögliche Nachweise genannt. Einschränkungen gibt es allerdings hinsichtlich der zeitlichen Gültigkeit. Diese wurde von verschiedenen Krankenkassen bis zu einem Alter zwischen 3 und 6 Monaten akzeptiert. Sofern kein solcher Nachweis vorliegt, muss der Patient diesen von einem Leistungserbringer einfordern, welcher das Vorliegen der entsprechenden Indikation explizit bescheinigen muss. Ein Kontakt zum Leistungserbringer wäre auch erforderlich, wenn nachträglich noch Kontraindikationen ausgeschlossen werden müssen:

*„[…] wenn eine Kontraindikation in der DiGA grundsätzlich auszuschließen ist, fordern wir Unterlagen an. Das muss nicht unbedingt eine Verordnung sein. Es wird dann natürlich häufig eine Verordnung, wenn der Patient dann zum Arzt geht und sagt ich muss einmal bestätigt bekommen, dass ich diese Kontraindikation nicht habe, dann kommt oft eine Verordnung zurück.“ (Krankenkasse)*

Dies führe dazu, dass der Prozess über den Selbstantrag häufig nicht flüssig laufe.

Teilweise äußerten Krankenkassen eine gewisse Skepsis, ob Patienten sich nicht in Versorgungslücken befinden, wenn sie den Weg des Selbstantrags einschlagen:

*„[…] wenn er jetzt bei uns aufschlägt und sagt, ich habe keine Verordnung, sondern ich mache mich gerade selbst schlau, ist die Frage warum. Hat der gerade eine Versorgungslücke, muss der lange auf einen Therapieplatz warten, hat der gar keinen, fühlt der sich im Regen stehen gelassen, […] können wir ihn überhaupt unterstützen jetzt auch bei der Suche nach einem Therapieplatz?“ (Krankenkasse)*

### Welche Vor- und Nachteile bringt dieser Zugangsweg mit sich?

#### Einstellung gegenüber dem Selbstantrag

Der Weg zur DiGA über die Genehmigung der Krankenkasse wird sowohl positiv als auch negativ bewertet (Vgl. Tab. 2 ). Für die direkte Beantragung von DiGA bei den Krankenkassen sprachen aus Sicht von Krankenkassen und Herstellern die Niederschwelligkeit des Zugangs sowie die größere Patientenautonomie. Durch den schnelleren und einfacheren Zugang könnten erneute Kontakte zum Arzt bzw. Psychotherapeuten und Wartezeiten vermieden werden. Insbesondere das Umgehen von Facharztterminen wurde seitens einer Krankenkasse als Vorteil gesehen. Ein Hersteller betonte, dass ein gewisser Grad an Patientenautonomie wünschenswert sei:

*„So, dann sollte ich doch in einem modernen Gesundheitswesen in der Lage sein, mich selbst zu kümmern und im Idealfall mit meiner Krankenkasse, die ja der Partner meines Vertrauens ist, zu sprechen […].“ (Hersteller)*

**Table:** **Tab. 2** Wahrgenommene Vor- und Nachteile des Selbstantrags.

Vorteile	Nachteile
Niederschwelliger Zugang Vermeiden von (Fach-) Arztbesuchen Vermeiden von Wartezeiten Größere Patientenautonomie	Verschiebung originärer Aufgaben von Ärzten zu Krankenkassen Aneignung der Therapiehoheit durch Krankenkassen Akzeptanzverlust von Ärzten und Psychotherapeuten gegenüber DiGA Ausbleibende Therapieüberwachung Gefahr einer unzureichenden klinischen Bewertung Mehraufwand für Krankenkassen durch Überprüfung von Indikationen

Neben den Vorteilen wurden aber auch innerhalb aller vertretenen Perspektiven Probleme beim selbstständigen Beantragen der DiGA gesehen. So wurde thematisiert, dass es einzuhaltende Standardzugangswege im Gesundheitssystem gäbe und eine Verschiebung originärer Aufgaben der Leistungserbringer hin zu den Krankenkassen problematisch sei. Ein weiterer Kritikpunkt lag in der ausbleibenden Therapieüberwachung.

Ein Hersteller plädierte für das Beibehalten von Standardzugangswegen im Gesundheitssystem und äußerte, dass das „Zwischenschalten“ von Krankenkassen zwischen Leistungserbringer und Patient nicht sinnvoll sei. Leistungserbringer verwiesen darauf, dass es aufgrund teilweise schwerer Diagnosen der gleichen Standards an Sorgfaltspflichten bedürfe.

Leistungserbringer und Hersteller sahen auch eine Gefahr darin, dass Krankenkassen sich durch den Selbstantrag die Therapiehoheit aneignen könnten und der Verlust dieser bei Leistungserbringern zu Akzeptanzverlust gegenüber DiGA führen könnte. Patientenvertreter und Leistungserbringer wiesen insbesondere darauf hin, dass es unklar sei, wie fundiert die der Krankenkasse vorliegende Diagnose ist. Die Möglichkeit, eine DiGA durch Selbstantragstellung auf der Basis eines bestehenden Diagnoseschlüssels zu erhalten, birgt die Gefahr einer unzureichenden klinischen Bewertung. Dies gilt insbesondere für die Abklärung von Dringlichkeit, Krisensituationen und Kontraindikationen wie Suizidalität oder Psychosen. Ohne eine sorgfältige fachliche Einordnung durch einen spezialisierten Leistungserbringer könnte eine DiGA daher auch an Personen gegeben werden, für die sie nicht geeignet oder sogar kontraindiziert ist. Eine Krankenkasse äußerte, dass es, da es ohnehin nötig sei, (Kontra-)Indikationen festzustellen, einfacher wäre, sich die DiGA direkt verordnen zu lassen.

*„[…] meistens ist es dann der Fall, dass dann der Arzt direkt eine Verordnung ausstellt, weil bevor er die Diagnose so bescheinigt und die Kontraindikation ausschließt, ist dann der einfachere Weg an der Stelle.“ (Krankenkasse)*

DiGA-Hersteller befürchteten einen Mehraufwand für Krankenkassen durch das Prüfen von (Kontra-)Indikationen. Zusätzlich würde Unsicherheit aufgrund von Diagnosen und Kontraindikationen seitens der Krankenkassen zu Zurückhaltung bei der Genehmigung führen.

Sowohl Leistungserbringer als auch Hersteller sahen ein Problem und eine Gefahr darin, dass Patienten alleingelassen werden. Es gäbe keinen klaren Ansprechpartner bei Schwierigkeiten. Die Notwendigkeit einer Therapieüberwachung wurde durch eine Parallele zur Arzneimittelversorgung dargestellt:

„Und wenn man sich so ein bisschen vorstellt, dass jemand mit einer Depression, wo zwar ärztlich oder psychotherapeutisch die Diagnose gesichert ist, zukünftig einfach in die Apotheke gehen könnte und sagen könnte, ich würde jetzt gerne ein Antidepressivum nehmen und zwar das da oben in der blauen Packung. Dann würden wir alle aus gutem Grund sagen, Moment, wir brauchen hier schon eine bessere Passung, nicht? Also es muss sich irgendwie dann doch noch einmal jemand die Patient*innen genau anschauen. Und das Wirkmittel angeschaut werden, was hier eingesetzt wird, um zu gucken, passt das für diese spezifischen Patient*innen. Und so sehen wir das bei den DiGA eigentlich auch.“ (Leistungserbringer)

DiGA-Hersteller befürchteten außerdem, dass Krankenkassen dazu neigen könnten, eigene (nicht als Medizinprodukt zertifizierte) Anwendungen statt der DiGA zu empfehlen.

Ein Experte ordnete die Thematik so ein, dass der Weg der Selbstbeantragung grundsätzlich richtig sei, die Vorteile aber durch die Umsetzung untergraben würden:

*„[…] In der Theorie halte ich diesen Zugangsweg nach wie vor für richtig, dass auch ganz ausdrücklich, aber nicht, wenn er so konterkariert wird, wie es die letzten Jahre der Fall war, dann ist es einfach Quatsch.“ (Hersteller)*

## Diskussion

Nicht nur das Konzept der DiGA, sondern auch der Zugangsweg der Selbstbeantragung ist neu für das Gesundheitswesen. Bislang war es so, dass für die von Krankenkassen im Leistungskatalog festgeschriebene Leistungen (z. B. Medikamente oder Hilfsmittel) zwangsläufig eine Verordnung durch einen Leistungserbringenden vorliegen musste, damit diese von der Krankenkasse finanziert werden. Mit der Einführung der DiGA wurden diese Standardzugangswege des Gesundheitswesens aufgebrochen und Patienten bekamen die Möglichkeit, sich selbstverantwortlich bei vorliegender Indikation Hilfe in Form von DiGA zu organisieren, für deren Kosten die Krankenkassen aufkommen (§ 33a SGB V).

Bislang spielt dieser Zugangsweg eine untergeordnete, aber nicht vernachlässigbare Rolle. So wird der Anteil der selbstbeantragten DiGA im letzten Bericht des GKV-Spitzenverbandes krankenkassenübergreifend mit gut 10% angegeben 3 . Während die Techniker Krankenkasse (TK) in ihrem DiGA-Bericht eine deutlich höhere Selbstbeantragungsquote von 18,2% angibt 12 , liegt der Wert bei der BARMER mit 9,6% darunter 5 . Auffällig ist, dass insbesondere bei psychischen Erkrankungen der Anteil an Selbstanträgen gering ausfällt: Depressionen (7,6%), Angststörungen (6,4%), Schlafstörungen (7,9%) und Tabakabhängigkeit (0,7%) 5 . Insgesamt stimmen die Angaben der befragten Experten mit den Ergebnissen aus (DiGA-)Berichten überein.

Es ist nachvollziehbar, dass Krankenkassen im Rahmen des Selbstantrags genau prüfen, aktuelle Indikationsnachweise verlangen und gegebenenfalls der Ausschluss von Kontraindikationen vorgelegt werden muss, da durch die Verlagerung der Therapiehoheit auch die damit verbundene Verantwortlichkeit von Leistungserbringenden (Verordnung) auf die Krankenkassen (Selbstantrag) übergeht. Insbesondere vor dem Hintergrund der Einführung des Digital-Gesetzes (DigiG) und der damit einhergehenden Ausweitung der Risikoklasse von IIa auf IIb gewinnt die Verlagerung der Therapiehoheit an Relevanz, da der Einsatz ein höheres Risiko birgt und somit einer therapeutischen Betreuung noch mehr Bedeutung zukommt. Beim Ausschluss von Kontraindikationen, insbesondere von Suizidgefahr, äußerten Leistungserbringende Skepsis, ob dies ausreichend fundiert geschieht.

Grundsätzlich birgt die Möglichkeit des Selbstantrags Vorteile. So kann dadurch zum einen die Patientenautonomie verbessert werden und zum anderen könnte ein niederschwelliger Zugang zum Versorgungsangebot DiGA geboten werden. Hiermit können insbesondere aktuell bestehende Zugangsprobleme zur ambulanten psychotherapeutischen Versorgung umgangen werden. In diesem Zusammenhang ist die weiterhin bestehende Stigmatisierung von psychischen Erkrankungen zu nennen, die Patienten hemmt, einen Psychotherapeuten aufzusuchen und eine Richtlinien-Psychotherapie zu beginnen. Insbesondere Männer, die an psychischen Erkrankungen leiden, könnten von diesem niederschwelligen Zugang profitieren, da diese bisher seltener eine psychotherapeutische Behandlung in Anspruch nehmen als Frauen 13 .

Einige Argumente sprechen aber auch gegen den Weg des Selbstantrags. Grundsätzlich sprechen sich Leistungserbringende sowie deren Fachgesellschaften für eine Einbindung der DiGA in die Versorgung und gegen einen davon losgelösten Einsatz aus. Die S3-Leitlinie zur Behandlung von Angststörungen rät sogar, im Fall von Panikstörungen oder Agoraphobie, von einer kognitiven Verhaltenstherapie-basierten Internetintervention als alleiniger Behandlungsmaßnahme ab 14 . Hybride Formen der Versorgung (digitale Interventionen kombiniert mit einer psychotherapeutischen Behandlung) erscheinen allerdings vielversprechend 15 .

Da die Krankenkassen eine DiGA genehmigen können, ohne eine hinreichende klinische Bewertung vorzunehmen, könnte dies die Akzeptanz und Kosteneffektivität erheblich beeinträchtigen. Ohne diese Informationen bleibt unklar, ob die DiGA für den Patienten wirklich geeignet ist und ob sie seine Therapieadhärenz in seiner spezifischen Situation fördert 16 .

Während es theoretisch Argumente für und wider den Selbstantrag gibt, können sich die möglichen Vorteile im Rahmen der aktuellen Ausgestaltung nicht durchsetzen. Die potenziell gewonnene Patientenautonomie und der niederschwellige Zugang werden dadurch aufgehoben, dass Patienten in der Regel darauf angewiesen sind, aktualisierte Indikationsnachweise oder Ausschlüsse von Kontraindikationen von Leistungserbringenden vorzuweisen. Durch die dadurch zwangsläufig notwendigen Kontakte zu Leistungserbringern stellt die Verordnung den einfacheren Weg dar.

Einschränkend sollte erwähnt werden, dass es nicht das Ziel der Studie war, eine vollständige Liste an Vor- und Nachteilen der Zugangswege zu erstellen, sondern vielfältige Perspektiven aufzuzeigen. Daher ist es unwahrscheinlich, dass für jede Stakeholdergruppe eine vollständige Datensättigung erreicht wurde. Weitere Forschung sollte sich auf spezielle Gruppen (z. B. Krankenkassen, Leistungserbringer, Patienten) konzentrieren. Im übergeordneten Forschungsprojekt DiGAPsy werden in weiteren Projektschritt insbesondere die Sichtweisen von Patienten und Leistungserbringern erfasst.

Um Patienten, die über ein ausgestelltes Rezept oder einen Indikationsnachweis verfügen, den Weg zur DiGA-Nutzung zu erleichtern, gibt es mittlerweile verschiedene Unterstützungsangebote (z. B. die Rezeptservices der DiGA-Hersteller). Hierbei können die Hersteller von Vertragsärzten schon ausgestellte DiGA-Rezepte bzw. Diagnosebestätigungen für die Versicherten bei der jeweils zuständigen Krankenkasse einreichen 17 . Diese wurden im Rahmen der Interviewstudie weder von Interviewenden noch von den Interviewten selber aufgegriffen.

## Fördermittel

Gemeinsamer Bundesausschuss — 01VSF22029

## Notes

Fazit Die beteiligten Stakeholdergruppen sollten ausführlich diskutieren, ob die Vorzüge oder Nachteile des Selbstantrags überwiegen. Sollte sich abschließend darauf verständigt werden, diesen Weg beizubehalten, wäre eine grundlegende Reform empfehlenswert. Der Gesetzgeber sollte einheitliche und eindeutige Kriterien (z. B. Art und Dauer der Gültigkeit von Indikationsnachweisen) festlegen, welche im Rahmen des Selbstantrags für alle Krankenkassenarten bindend sind. Es sollte darauf geachtet werden, dass die Gradwanderung zwischen Prüfung der klinischen Notwendigkeit einerseits und Gewährung von Patientenautonomie andererseits gelingt. Die Kriterien sollten für Patienten nachvollziehbar und leicht verständlich und für die Krankenkassen bindend sein.

## Zusätzliches Material

Zusätzliches Material Zusätzliches Material
