# Supplementary material for: Access to Digital Health Applications – Prescription by Physicians vs Patient Requests Made Directly to Health Insurance Companies: Interviews with Experts
Source: Gesundheitswesen. 2026 Jun 3;88(7):455–61. [Article in German] doi: 10.1055/a-2800-1191 (PMC13345681; doi:10.1055/a-2800-1191)
Supplement: Supplementary file 1 — Zusätzliches Material [file 10-1055-a-2800-1191-2025-04-2254-oa.pdf]

Appendix 1. Mit den Experten besprochene Themen sowie Gesprächsleitfäden

	Leistungs-erbringer	Patienten	DiGA-Hersteller	Kranken-kassen
**Einleitung**	x	x	x	x
**Einsatz**		x		
**Genehmigungsprozess**	x		x	x
**Verschreibungsprozess**	x	x	x	x
**Hürden**	x	x	x	x
**Einbindung**	x	x	x	x
**Informationen**	x	x	x	x
**Zulassungsprozess**	x		x	

Das Gesundheitswesen | DOI 10.1055/a-2800-1191 | © The Author(s).
